# Recovering Bioactive Compounds from Olive Oil Filter Cake by Advanced Extraction Techniques

**DOI:** 10.3390/ijms150916270

**Published:** 2014-09-15

**Authors:** Jesús Lozano-Sánchez, María Castro-Puyana, Jose A. Mendiola, Antonio Segura-Carretero, Alejandro Cifuentes, Elena Ibáñez

**Affiliations:** 1 Research and Development Functional Olive Oil Department, Aceites Maeva S.L., Avda. Incar, S/N 18130 Escúzar Granada, Granada 18007, Spain; E-Mail: jesusls@ugr.es; 2 Research and Development of Functional Food Centre, Health Science Technological Park, Avda. del Conocimiento, Ed. BioRegión, Armilla, Granada 18007, Spain; E-Mail: ansegura@ugr.es; 3 Laboratory of Foodomics, Institute of Food Science Research (CIAL-CSIC), Nicolás Cabrera 9, Campus Cantoblanco, Madrid 28049, Spain; E-Mails: maria.castrop@uah.es (M.C.-P.); j.mendiola@csic.es (J.A.-M.); elena.ibanez@csic.es (E.I.); 4 Department of Analytical Chemistry, Physical Chemistry and Chemical Engineering, Faculty of Biology, Environmental Science and Chemistry, University of Alcalá. Ctra. Madrid-Barcelona, Km. 33.600, 28871, Alcalá de Henares, Madrid 28049, Spain

**Keywords:** olive oil by-products, filter cake, phenolic compounds, pressurized liquid extraction, HPLC-ESI-TOF/MS, GRAS solvents

## Abstract

The potential of by-products generated during extra-virgin olive oil (EVOO) filtration as a natural source of phenolic compounds (with demonstrated bioactivity) has been evaluated using pressurized liquid extraction (PLE) and considering mixtures of two GRAS (generally recognized as safe) solvents (ethanol and water) at temperatures ranging from 40 to 175 °C. The extracts were characterized by high-performance liquid chromatography (HPLC) coupled to diode array detection (DAD) and electrospray time-of-flight mass spectrometry (HPLC-DAD-ESI-TOF/MS) to determine the phenolic-composition of the filter cake. The best isolation procedure to extract the phenolic fraction from the filter cake was accomplished using ethanol and water (50:50, *v*/*v*) at 120 °C. The main phenolic compounds identified in the samples were characterized as phenolic alcohols or derivatives (hydroxytyrosol and its oxidation product), secoiridoids (decarboxymethylated and hydroxylated forms of oleuropein and ligstroside aglycones), flavones (luteolin and apigenin) and elenolic acid derivatives. The PLE extraction process can be applied to produce enriched extracts with applications as bioactive food ingredients, as well as nutraceuticals.

## 1. Introduction

Olive oil production has a great economic impact for many Mediterranean countries that comprise more than 95% of the world olive-oil production. Moreover, 75% is being produced by Mediterranean Member States of the European Union [1]. Extra-virgin olive oil (EVOO) is produced from the olive fruit exclusively by mechanical or other physical means under conditions that do not lead to alterations in the oil [2]. This production process preserves the oil’s characteristic flavor, as well as its nutritional and health benefits. It is widely accepted that the consumption of EVOO is associated with a low incidence of cardiovascular diseases, neurological disorders and breast cancer, among others [3]. These health properties have been related to its chemical composition, including major components, such as triacylglycerols, that represent more than 98% of the total oil weight, and minor components, which are present in very low amounts (about 2% of oil weight) and comprise more than 300 different chemical compounds [4], including polyphenols with important associated bioactivities [5,6].

Phenolic compounds represent an important class of natural antioxidants. The term “phenolic compound” includes a large number of secondary plant metabolites that differ in chemical structure and reactivity, ranging from simple compounds to highly polymerized compounds. The phenolic composition of EVOO is influenced by complex multivariate interactions from genotype, agricultural, environmental and technological factors [7]. The steps in the production process include harvesting, washing and crushing of olives, malaxation of olive paste, centrifugation, storage and filtration. These steps influence the final qualitative and quantitative phenolic composition of olive oil. The loss of polyphenols during the production process is related to its partitioning between oil and by-products. EVOO production is associated with the generation of large quantities of wastes (olive-oil-mill wastewater (OMWW), olive pomace, storage by-products and filter cake), which could be an alternative source of these compounds [8]. In a previous work from our laboratory, we evaluated the effect of filter aids (organic or inorganic) on the retention of phenolic compounds from EVOO [9], demonstrating that, depending on the nature of the filter, substantial amounts of phenolic compounds were retained, thus becoming an interesting source of these compounds.

In recent years, interest in EVOO by-products as natural sources of polyphenols has increased as a way to revalorize such by-products, while minimizing the environmental impact associated with its disposal. Different pilot-plant and industrial processes have been employed to obtain polyphenol-enriched extracts, mainly in hydroxytyrosol, from OMWW and olive-oil-milling waste (*alperujo*). The main processes proposed to recover the phenolic compounds from by-products include resin chromatography, microfiltration, ultrafiltration, nanofiltration and reverse osmosis, solid-liquid or liquid-liquid solvent extractions [8,9,10,11,12,13,14]. However, more research is needed to produce purified antioxidant extracts rich in hydroxytyrosol and other possible bioactive compounds from EVOO by-products, e.g., by using more selective and environmentally-friendly extraction techniques, such as supercritical fluid extraction (SFE) and pressurized liquid extraction (PLE). These extraction processes provide several advantages compared to the conventional ones, mainly related to higher selectivity, shorter extraction times and lower consumption of toxic organic solvents [15]. Although different olive by-products have been extracted by using these new processes, for example, residues of the olive oil deodorization process [16], olive cake [17] or olive leaves using hexane and supercritical CO_2_ or PLE [18,19], more work is clearly needed about the use of PLE to extract valuable compounds from olive filter-cakes, mainly considering that the recovery of bioactive compounds from olive waste products provided very good results using green solvents, such as water or ethanol [20]. Moreover, the possibility of using advanced chromatographic techniques coupled to different detectors, especially mass spectrometers, makes it possible to identify compounds responsible for the associated bioactivities; indeed, several studies have already investigated the chemical composition of olive oil by-products [9,17,19,21,22].

Because the antioxidant and healthy properties of olive polyphenols have attracted much scientific interest, the aim of this work is to develop a PLE process followed by the analytical characterization using high-performance liquid chromatography coupled to diode array detection and electrospray time-of-flight mass spectrometry (HPLC-DAD-ESI-TOF/MS) to determine the phenolic composition of purified filter-cake extracts with future applications in the development of functional foods, as well as nutraceuticals.

## 2. Results and Discussion

To study the capabilities of PLE to extract phenolic compounds from filter cakes, two food-grade solvents categorized as generally recognized as safe (GRAS), such as water and ethanol, were investigated. As can be observed in Table 1, different combinations of solvent composition and temperatures were tested to optimize the extraction of phenolic compounds from filter cake. The percentages of ethanol in the mixture ethanol:water from zero to 85% and temperatures between 40 and 175 °C were used in order to cover a wide range of dielectric constants from 21 (when ethanol:water 85:15 *v*/*v* at 175 °C was used) to 59.1 (using ethanol:water 15:85 *v*/*v* at 60 °C), covering, in this way, a wide range of polarities. The extraction temperatures were chosen above (120 and 175 °C) and below (40 and 63 °C) the boiling point of both solvents. It is worth highlighting that 175 °C was the maximum temperature at which the experiments could be performed without experimental problems (e.g., blockage of the lines of the PLE instrument). In addition, to carry out an efficient extraction of phenolic compounds, it was mandatory to eliminate previously the lipophilic fraction from the filter cake. To do that, 8 g of filter cake were loaded in the extraction cell, and a clean-up step before the PLE extraction was performed. As Table 1 shows, a high percentage (around 50%) of the lipophilic fraction was retained in the filter cake loaded in the extraction cell. Once this fraction was eliminated, the influence of the extraction temperature and solvent composition on the extraction yield (determined as dry weight/amount of sample in the cell after the clean-up step) were evaluated. Even though is not possible to achieve high extraction yields, the data obtained demonstrated that the PLE extraction provides the highest extraction yield (5.8%) when a temperature of 120 °C and a composition of ethanol:water 50:50 *v*/*v* were employed, *i.e*., a solvent composition with an intermediate polarity (a dielectric constant of 34.7).

**Table 1:** Extraction conditions, dielectric constant and extraction yield obtained from the pressurized liquid extraction (PLE) extraction of filter cake (to carry out the PLE, 8 g of filter cake were loaded in the extraction cell).

T (°C)	Ethanol in the Mixture Ethanol:Water (%)	Dielectric Constant in the Mixture Ethanol:Water	Amount of Lipophilic Fraction Eliminated in the Clean-Up Step (g)	Amount of Sample in the Cell after Clean-Step (g)	Extraction Yield (%)
40	50	48.0	4.789	3.237	3.0
175	85	21.6	4.823	3.192	4.3
60	15	59.1	4.850	3.159	5.6
120	0	50.4	4.129	3.887	3.5
120	50	34.7	4.936	3.080	5.8

**Figure 1:**
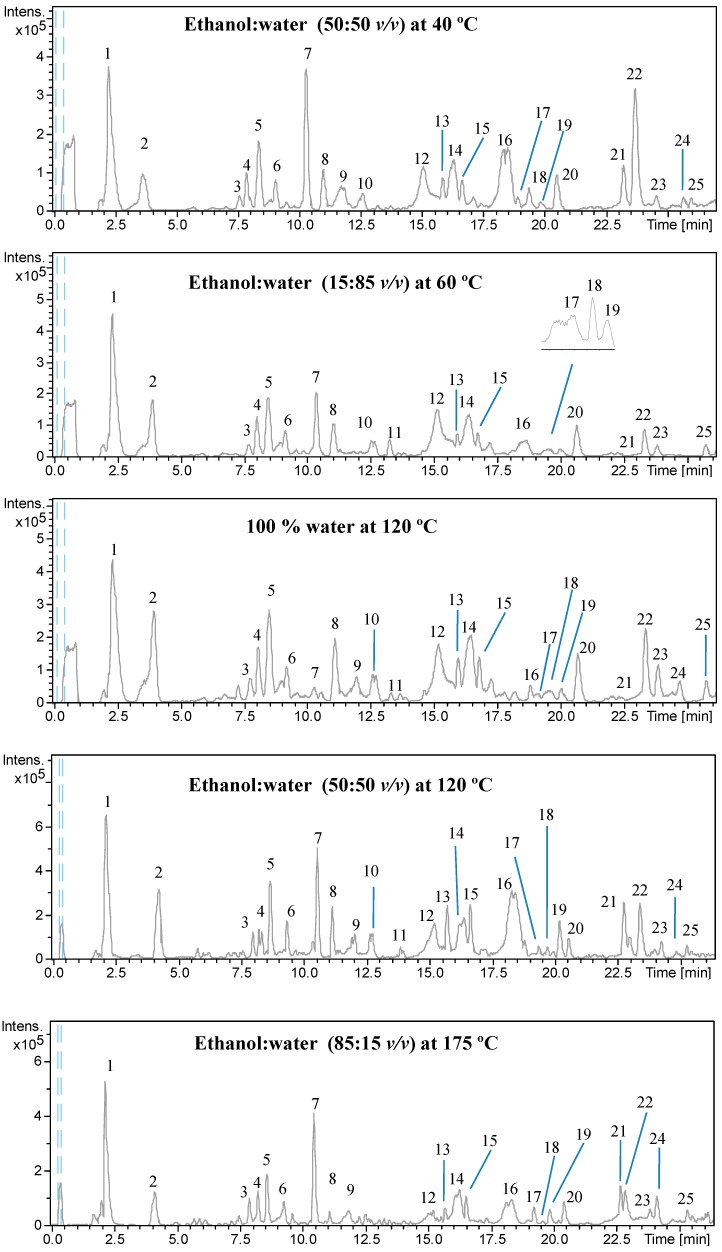
Base peak chromatogram (BPC) obtained from the high-performance liquid chromatography coupled to diode array detection and electrospray time-of-flight mass spectrometry (HPLC-DAD-ESI-TOF/MS) analysis of the filter cake extracts obtained under different PLE extraction conditions. Peaks have been numbered according to the elution order. For the peak identification, see Table 2. Chromatographic conditions are described in the Experimental Section.

To evaluate the differences among the different extraction conditions, all the filter cake extracts were analyzed by HPLC-DAD-ESI-TOF/MS in order to establish the differences in their phenolic composition. Figure 1 depicts the chromatographic profiles obtained under the extraction conditions described in Table 1. Basically, all of the filter cake extracts show similar profiles, making it possible to identify up to 25 compounds (*vide infra*). However, some differences can be observed in the peak intensity of several compounds depending on the extraction conditions. For instance, by comparing the chromatogram of the extract obtained at 120 °C using 100% water with the extract obtained using the same temperature (120 °C), but using ethanol:water, 50:50 (*v*:*v*), it can be seen that some compounds (e.g., Peak 20) are better extracted using 100% water, while other compounds (e.g., Peaks 7, 16, 21) are better extracted using ethanol:water 50:50. This result shows the good possibilities of PLE in terms of selectivity variation.

The identification of the phenolic compounds was attained combining their retention times, the information provided by the two detectors used in this work (DAD and MS) and data previously reported in the literature. Table 2 summarizes the compounds identified, including their retention time, the molecular formula generated by the TOF-MS analyzer, the mass error (ppm), the comparison between the theoretical and the experimental isotopic pattern (given as the σ-value) and the wavelength of the maximum of the ultraviolet-visible (UV–Vis) spectra. Although nine compounds remain unknown (Uk) (indicated as Uk 1–9), sixteen compounds were identified. Among them, two were characterized as phenolic alcohols or derivatives, five were identified as elenolic acid or derivatives, six were secoiridoids, two were characterized as flavones and one compound was identified as quinic acid (Peak 1), which is not a phenolic molecule.

**Table 2:** Tentative identification of phenolic compounds and their derivatives in the filter cake PLE extracts.

Peak	Tentative Identification	Molecular Formula	*R*_t_ (min)	[M–H]^−^ cal	[M–H]^−^ exp	Error (ppm)	σ-Value	λ_max_ (nm)	References
**1**	Quinic acid	C_7_H_12_O_6_	2.1	191.0561	191.0561	0.1	10.4		[21,22]
**2**	Oxidized hydroxytyrosol	C_8_H_8_O_3_	3.9	151.0401	151.0408	5.2	4.2	232, 280	[21,22]
**3**	Uk 1	C_14_H_20_O_8_	8.0	315.1085	315.1098	−4.0	0.9	284, 354	
**4**	Hydroxytyrosol	C_8_H_10_O_3_	8.3	153.0557	153.0568	7.4	13.3	232, 280	[9,17,19,21,22,23,24,25]
**5**	Uk 2	C_17_H_28_O_11_	8.6	407.1559	407.1595	−8.8	8.2	280, 320	
**6**	Hydroxylated product of the dialdehydic form of decarboxymethyl elenolic acid	C_9_H_12_O_5_	9.2	199.0612	199.0625	−6.6	4.2	240	[21,22]
**7**	Uk 3	C_8_H_8_O_3_	10.5	151.0401	151.0408	−6.8	10.5	240, 280	
**8**	Dialdehydic form of decarboxymethyl elenolic acid	C_9_H_12_O_4_	11.0	183.0663	183.0783	−7.0	8.8	240	[21,22,23]
**9**	Uk 4	C_17_H_20_O_8_	12.0	351.1085	351.1090	−1.2	6.7	233, 288	
**10**	Uk 5	C_16_H_26_O_10_	12.7	377.1453	377.1457	−6.4	4.2	266	
**11**	Hydroxylated form of elenolic acid I	C_11_H_14_O_7_	13.8	257.0667	257.0687	−8.0	1.4	240	[21,22,25]
**12**	Elenolic acid	C_11_H_14_O_6_	15.2	241.0718	241.0740	−9.1	3.0	240	[9,21,22,23,24,25]
**13**	Hydroxylated form of elenolic acid II	C_11_H_14_O_7_	15.7	257. 0667	257.0689	−8.5	1.2	240	[21,22]
**14**	Decarboxymethyl-oleuropein aglycone	C_17_H_20_O_6_	16.3	319.1187	319.1206	−6.0	1.3	230, 280	[9,17,21,22,24,25]
**15**	Hydroxy-decarboxymethyl oleuropein aglycone	C_17_H_20_O_7_	16.9	335.1136	335.1170	−9.2	1.1	230, 280	[9,21,22,24,25]
**16**	Uk 6	C_19_H_24_O_7_	18.2	363.1449	363.1466	−4.6	8.4	255	
**17**	Decarboxymethyl-ligstroside aglycone	C_17_H_20_O_5_	19.3	303.1238	303.1238	0.0	20.5	230, 280	[17,21,22,24,25,26]
**18**	Hydroxy-decarboxymethyl-ligstroside aglycone	C_17_H_20_O_6_	19.6	319.1187	319.1195	−2.4	3.7	230, 280	[21,22,23,24]
**19**	Uk 7	C_25_H_28_O_13_	20.1	535.1457	535.1463	−1.1	3.9	277, 330	
**20**	Uk 8	C_42_H_74_O_10_	20.6	737.5209	737.5219	−1.4	29.9	331	
**21**	10-Hydroxy-oleuropein aglycone	C_19_H_22_O_9_	22.7	393.1191	393.1216	−6.3	4.7	236, 282	[9,21,24]
**22**	Luteolin	C_15_H_10_O_6_	23.8	285.0405	285.0420	−5.4	2.0	254, 348	[9,17,21,22,23,24,25]
**23**	Oleuropein aglycone or isomer	C_19_H_22_O_8_	24.1	377.1242	377.1249	−2.5	0.3	236, 282	[9,17,21,22,23,24,25]
**24**	Uk 9	C_21_H_26_O_9_	24.7	421.1504	421.1539	−8.2	10.6	255	
**25**	Apigenin	C_15_H_10_O_5_	25.6	269.0455	269.0480	−9.0	12.5	268, 338	[9,17,21,22,23,24,25]

Unknown compounds (Uk).

The oxidation product of hydroxytyrosol (Peak 2) and hydroxytyrosol (Peak 4), whose [M–H]^−^ are 151.0408 and 153.0568 *m*/*z*, respectively, were identified as phenolic alcohols in the filter cake extract. Both compounds have been previously described in olive oils by-products [9,17,19,21,22]. Several polar compounds derived from elenolic acid (Peak 12, with 241.0740 *m*/*z*) were also identified. Thus, different peaks were assigned to the hydroxylated product of dialdehydic form of decarboxymethyl-elenolic acid (Peak 6), the dialdehydic form of decarboxymethyl-elenolic acid (Peak 8), the hydroxylated form of elenolic acid I (Peak 11) and the hydroxylated form of elenolic acid II (Peak 13). The presence of all these elenolic-acid and phenolic alcohol derivatives could be related to the hydrolysis of secoiridoids, followed by several reactions, such as oxidation, hydration and loss of the carboxylic and carboxymethyl groups [17,21,22]. Indeed, secoiridoids in their aglyconic forms, the main compounds in EVOO, are characterized by the presence of elenolic acid derivatives bonded to a phenolic alcohol structure. Hydrolysis of the ester bond between the phenolic portion and the rest of the molecule could explain the origin of the free acidic and alcoholic moieties.

**Figure 2:**
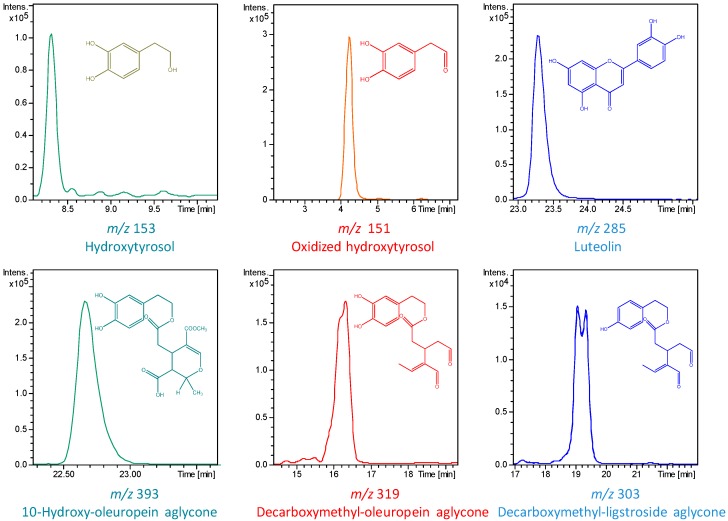
Extracted ion chromatograms (EIC) of some phenolic compounds characterized by high-performance liquid chromatography electrospray time-of-flight mass spectrometry (HPLC-ESI-TOF/MS).

The chemical precursors of these compounds have also been detected in the samples analyzed: oleuropein aglycone (Peak 23) and its hydroxyl and/or decarboxymethyl derivatives (Peaks 14, 15 and 21), as well as ligstroside aglycone decarboxymethyl and hydroxydy-decarboxymethyl derivatives (Peaks 17 and 18). These phenolic compounds have been previously reported as characteristic polyphenols in EVOO [22,23,24,25]. Finally, the molecular ions at 285.0420 *m*/*z* and 269.0480 *m*/*z* were assigned to flavones, namely luteolin (Peak 22) and apigenin (Peak 25). Some structures along with the extracted ion chromatogram of the main polyphenols compounds identified in the filter cake extract are shown in Figure 2.

**Figure 3:**
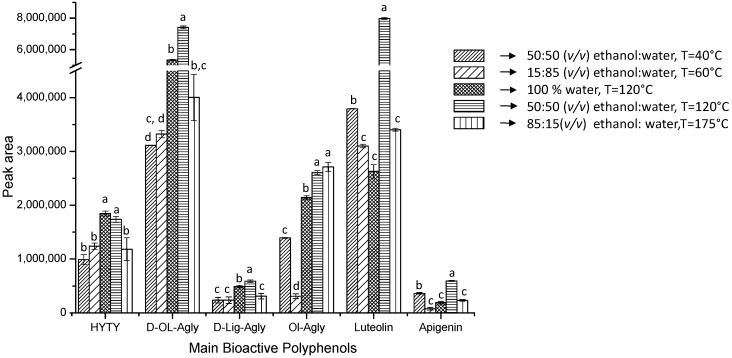
Peak area of the main bioactive phenolic compounds characterized in filter cake (HYTY, hydroxytyrosol; D-OL-Agly, decarboxymethyl-oleuropein aglycone; D-Lig-Agly, decarboxymethyl-ligstroside aglycone; Ol-Agly, oleuropein aglycone; luteolin and apigenin) under different PLE conditions. Values with the same letter (a, b, c or d) show that there is no statistically significant difference between the area of these compounds at the 95% confidence level (*p* ≤ 0.05).

An additional proof of the different selectivity that PLE can provide is obtained comparing the main phenolic content, expressed as the peak area, from the different PLE extracts. Figure 3 shows the peak area obtained for the main bioactive compounds characterized in the filter cake extracts (hydroxytyrosol, decarboxymethyl-oleuropein aglycone, decarboxymethyl-ligstroside aglycone, oleuropein aglycone, luteolin and apigenin) under the different extraction conditions studied in this work. In addition, an analysis of variance (ANOVA) at the 95% confidence level was performed to analyze statistically significant differences among the areas of these compounds. The results obtained showed that PLE extraction using 100% water at 120 °C, 50:50 (*v*/*v*) ethanol:water at 120 °C and 85:15 (*v*/*v*) ethanol:water at 176 °C are the most appropriate for extracting the compounds selected. The peak area obtained for hydroxytyrosol and decarboxymethyl-ligstroside aglycone was slightly higher when the extraction was carried out using 100% water at 120 °C; however, for all of the other compounds, the most effective extraction was achieved employing 50:50 (*v*/*v*) ethanol:water at 120 °C. This result combined with the fact that these conditions gave the highest extraction yield (see Table 1) demonstrate that the PLE methodology developed in this work, based on the use of ethanol:water (50:50 *v*/*v*) as the extraction solvent at 120 °C, is suitable for extracting phenolic compounds from filter cake.

Conventional systems to extract phenolic compounds from olive by-products are based on different chemical/physical procedures using organic solvents. In general, these processes include adjusting the pH value, enzymatic hydrolysis, membrane technologies, thermal evaporation and recovering organic solvents using adsorbent resins [8,9,10,11,12,13,14]. These technologies have been addressed for reducing the organic matter and polyphenols to detoxify their effects on the environment. Although diverse combinations of these processes have been applied, their potential for recovering all EVOO polyphenols from olive wastes is still doubtful. These systems need successive stages to gradually increase the purity of the final products and have been mainly focused on obtaining hydroxytyrosol-rich extracts.

However, the well-known bioactivity of other olive polyphenols has stimulated the development of new green processes for recovering all of these compounds. PLE is more suitable for extracting compounds with a wider range of polarity than conventional systems. Likewise, the recovery of bioactive polyphenol from other olive by-products has been developed using green technologies [18,19]; the proposed extraction methodology based on PLE provides natural extracts from filter cake enriched in polyphenols with healthy benefits. It is interesting to mention that although the action mechanism of these bioactive compounds could have different cellular targets and synergic effects, the bioactivity of these molecules in chronic diseases seems to be mainly related to their antioxidant activity. Indeed, hydroxytyrosol and secoiridoids, mainly decarboxymethyl-oleuropein aglycone (also known as oleacein) and decarboxymethyl-ligstroside aglycone (or oleocanthal), have higher antioxidant activity than other phenols, linked to their o-diphenolic structure. Several studies have reported the application of these compounds against cancer [27,28,29], inflammation [26,30,31], atherosclerosis and metabolic syndrome [32,33], which makes the use of PLE methods, such as the one proposed in this work, especially interesting for the extraction of this type of compound.

## 3. Experimental Section

### 3.1. Samples

EVOOs were produced at the Maeva production plant (Aceites Maeva S.L., Granada, Spain) in September 2012. Olives (Picual variety) were processed by a continuous industrial plant equipped with a hammer crusher, a horizontal malaxator and a two-phase decanter. To remove suspended solids and reduce moisture, 5000 L of cloudy olive oil were filtered at room temperature under a constant flow using an industrial filtration equipment based on a filter tank in conjunction with 50 kg of cellulose fiber as a filter aid (Vitacel^®^L90, Rettenmaier Ibérica, Barcelona, Spain). After filtration, the olive oil was directly transferred from the filter tank to the bottling equipment, and the filter cake was collected. To achieve representative results and eliminate factors that could affect the phenolic profile, this fraction was isolated from the filter cake without the storage of wastes.

### 3.2. Chemicals

All chemicals were of analytical reagent grade. Methanol, ethanol and *n*-hexane were purchased from Merck (Darmstadt, Germany). Acetic acid was purchased from Fluka, Sigma–Aldrich (Steinheim, Germany). Double-deionized water with conductivity less than 18.2 MΩ was obtained with a Milli-Q system (Millipore, Bedford, MA, USA).

### 3.3. Pressurized Liquid Extraction

Extractions of phenolic and polar compounds from the filter cake were performed using an accelerated solvent extractor (ASE 200, Dionex, Sunnyvale, CA, USA) equipped with a solvent controller. Extractions were done using different combinations between solvent composition and temperatures to cover a wide range of dielectric constants. Thus, percentages of ethanol in the mixture ethanol:water from 0% to 85% and temperatures above (120 and 175 °C) and below (40 and 63 °C) the boiling point of both solvent were tested.

Prior to use, solvents were sonicated for 15 min to avoid any possible oxidation and to remove the dissolved oxygen. All extractions were done using 11-mL extraction cells, containing 8 g of filter cake mixed homogeneously with 2.5 g of sand. A preliminary extraction (clean-step) based on the use of *n*-hexane as the solvent and 1500 psi and room temperature as the experimental conditions was carried out to remove the lipophilic fraction from the filter cake. After that, the extraction of phenolic compounds was performed according to the extraction conditions mentioned above combined with 1500 psi and 20 min as the pressure and extraction time. An extraction cell heat-up step was carried out for a given time fixed by the system prior to any extraction (the warming up time ranged from 5 to 9 min depending on the extraction temperature).

The extract obtained was protected from light and stored at −20 °C until dried. For ethanol evaporation, a rotavapor R-210 from Büchi Labortechnik AG (Flawil, Switzerland) was used, whereas for water evaporation, a freeze-dryer (Virtis Unitop 400 SL, Gardiner, NY, USA) was employed.

### 3.4. High-Performance Liquid Chromatography Coupled to Diode Array Detection and Electrospray Time-of-Flight Mass Spectrometry (HPLC-DAD-ESI-TOF/MS) Analysis

High-performance resolution liquid chromatography (HPLC) coupled to a diode-array-detector (DAD) and an electrospray time-of-flight mass spectrometry (ESI-TOF-MS) was used to characterize the phenolic profile of extracts using an Agilent 1200-RRLC system (Agilent Technologies, Waldbronn, Germany) equipped with a vacuum degasser, autosampler and a binary pump. The analytical column used was a 150 mm × 4.6 mm internal diameter , 1.8-μm, Zorbax Eclipse Plus C18 (Agilent Technologies, Palo Alto, CA, USA). The flow rate was 0.80 mL/min, and the temperature of the column was maintained at 25 °C. The mobile phase was a mixture of water with 0.25% (Solvent A) and methanol (Solvent B) eluted according to the following multistep gradient reported in a previous study [24]: 0 min, 5% Solvent B; 7 min, 35% Solvent B; 12 min, 45% Solvent B; 17 min, 50% Solvent B; 22 min, 60% Solvent B; 25 min, 95% Solvent B; 27 min, 5% Solvent B and, finally, a conditioning cycle of 5 min with the same conditions for the next analysis. The injection volume was 10 µL. The compounds separated were monitored in sequence first with DAD (240 and 280 nm), and then, MS was performed using the microTOF (Bruker Daltonik, Bremen, Germany), which was coupled to the HPLC system. At this stage, the use of a splitter was required for the coupling with the MS detector, as the flow arriving to the TOF detector had to be 0.2 mL/min in order to ensure reproducible results and a stable spray. The TOF mass spectrometer was equipped with a model G1607A ESI interface (Agilent Technologies, Palo Alto, CA, USA) operating in negative ion mode. External mass spectrometer calibration was performed with sodium acetate clusters (5 mM sodium hydroxide in water/2-propanol 1/1 (*v*/*v*), with 0.2% of acetic acid) in quadratic þ high-precision calibration (HPC) regression mode.

The calibration solution was injected at the beginning of the run, and all of the spectra were calibrated prior to polyphenol identification. The optimum values of the source and transfer parameters were used for good sensitivity and a reasonable resolution of the mass range for compounds of interest (*m*/*z* 50–1000) in order to improve ionization performance [24]. The accurate mass data for the molecular ions were processed using the software, Data Analysis 3.4 (Bruker Daltonik), which was provided with a list of possible elemental formulas by using the Generate Molecular Formula Editor. The latter uses a carbon, hydrogen, nitrogen and oxygen (CHNO) algorithm that provides standard functionalities, such as minimum/maximum elemental range, electron configuration and ring-plus double-bond equivalent, as well as a sophisticated comparison of the theoretical with the measured isotopic pattern (σ-value) for increased confidence in the suggested molecular formula. The widely-accepted accuracy threshold for confirmation of elemental compositions was established at 5 ppm for most of the compounds.

### 3.5. Statistical Analysis

One-way analysis of variance (ANOVA) at a 95% confidence level (*p* ≤ 0.05) was performed to analyze statistically significant differences among the areas of the main phenolic compounds extracted. To do that, Origin (Version Origin Pro 8 SR0, Northampton, MA, USA) was employed.

## 4. Conclusions

The proposed PLE methodology based on the use of a mixture of two GRAS solvents, such as ethanol and water at 120 °C, has shown great potential to extract phenolic compounds (with healthy properties) from filter cake, a by-product generated during EVOO filtration. The phenolic composition of the extracts from filter cake was characterized by using HPLC-DAD-ESI-TOF-MS. Thus, the main compounds found in the extract were identified as phenolic alcohols or derivatives (hydroxytyrosol and its oxidation product), secoiridoids (decarboxymethylated and hydroxylated forms of oleuropein and ligstroside aglycones), flavones (luteolin and apigenin) and elenolic acid derivatives. The results from this work demonstrate that the PLE protocol developed could be considered as a fast and environmental-friendly extraction process to produce enriched phenolic extracts from filter cake with applications as bioactive food ingredients and nutraceuticals.
